# Neuromedin B mediates IL-6 and COX-2 expression through NF-κB/P65 and AP-1/C-JUN activation in human primary myometrial cells

**DOI:** 10.1042/BSR20192139

**Published:** 2019-10-11

**Authors:** Texuan Zhu, Jingfei Chen, Yanhua Zhao, Jiejie Zhang, Qiaozhen Peng, Jingrui Huang, Jiefeng Luo, Weishe Zhang

**Affiliations:** 1 Department of Obstetrics and Gynecology, Xiangya Hospital, Central South University, Changsha 410008, Hunan, China; 2 Hunan Engineering Research Center of Early Life Development and Disease Prevention, Hunan, China

**Keywords:** AP-1/c-Jun, COX-2, human primary myometrial cells, IL-6, neuromedin B, NF-κB/p65

## Abstract

Neuromedin B (NMB) and its receptor regulate labor onset by mediating inflammatory factors; however the underlying mechanisms remain poorly understood. The present study is aimed to investigate the mechanisms of NMB-induced cyclo-oxygenase 2 (COX-2) expression and interleukin (IL)-6 generation in human primary myometrial cells. The results indicated that NMB could increase phosphorylation of nuclear factor κB (NF-κB) transcription factor p65 (p65) and Jun proto-oncogene, activator protein 1 (AP-1) transcription factor subunit (c-Jun), and in turn, markedly up-regulated the expression levels of COX-2 and IL-6. This up-regulation was significantly attenuated by knockdown of p65 or c-Jun, and enhanced by overexpression of p65 or c-Jun. Furthermore, we identified a potential interaction between p65 and c-Jun following NMB stimulation. In addition, a significant positive correlation was observed between the amount of phosphorylated p65 and the levels of COX-2 and IL-6, and between the amount of phosphorylated c-Jun and COX-2 and IL-6 levels. These data suggested that NMB-induced COX-2 and IL-6 expression were mediated via p65 and c-Jun activation.

## Introduction

Preterm birth (PTB), defined as birth before 37 weeks, is the leading cause of perinatal morbidity and mortality worldwide [1]. It is estimated that over 41000 preterm infants are born each day across the world [1]. Preterm neonates are at a high risk of long-term disabilities, including mental retardation, cerebral palsy, lung disease, and hearing loss [2]. However, the precise mechanism controlling labor onset in humans remains unclear.

The key approach to women with suspected preterm labor continues to be the inhibition of uterine contractions. This focus on contraction inhibition has led to the development of agents affecting myometrial contractility [3]. Tocolysis is currently considered as an important strategy for PTB because it can inhibit uterine contractility to prolong pregnancy. There are a variety of tocolytic agents in current use, which act on the uterus in varying ways. The most common are calcium channel blockers, betamimetics, magnesium sulfate, cyclo-oxygenase (COX) inhibitors, oxytocin agonists, and nitric oxide. However, their lack of long-term efficacy, and maternal and fetal side effects, make them clinically unsatisfactory [4]. Thus, investigation of novel targets associated with uterine contraction might provide new insights into the prevention and treatment of PTB. Through our initial cDNA microarray screening, we found that the mRNA encoding neuromedin B (NMB) receptor (*NMBR*), but not the oxytocin receptor (OTR) or β_2_-adrenergic receptor, was differentially expressed in the human uterine myometrium during spontaneous or oxytocin-induced labor [5]. The levels of *Nmbr* mRNA and protein peaks at parturition and decreases sharply after delivery in mouse myometrial cells [6,7]. The NMBR agonist, NMB, selectively binds to NMBR to mediate many biological effects, such as contraction of uterine smooth muscle [7], as well as urogenital and gastrointestinal smooth muscles [8]. Maternal exposure to the NMB shortened the gestational age of mice [7]. All the above suggested that NMBR is likely to be an ideal candidate target in PTB. However, the specific mechanisms of NMB/NMBR in the regulation of labor onset remain to be determined.

The transcription factor nuclear factor κB (NF-κB) is known to play a fundamental role in a number of physiological processes. In resting cells, NF-κB is sequestered in the cytoplasm through direct interaction with a member of the IκB family of inhibitor proteins such as IκBα. Various stimuli could lead to the activation of the IKK complex which contains two IκB kinases, IKKα and IKKβ. Phosphorylation of IκBα by the IKK complex leads to its polyubiquitination and subsequent degradation. The liberated NF-κB dimer then translocates to the nucleus where it recognizes and binds specific DNA sequences termed as κB sites [9]. WIP1 phosphatases, a member of the Ser/Thr PP2C family, could suppress phosphorylation of p65 resulting in its inactivation [10]. Accumulating evidence demonstrated that NF-κB transcription factor p65 (p65) takes an active part in labor onset by regulating a variety of cytokines, including interleukin (IL)-6, type-2 COX enzyme (COX-2), IL-8, IL-1β, matrix metalloproteinase (MMP)-9 and tumor necrosis factor α (TNF-α) [11–18]. Our previous study found that NMB could activate p65 and induce the expression of IL-6 to control the free [Ca^2+^]_i_ in mice myometrial cells [19]; but a higher level of inhibition of [Ca^2+^]_i_ was detected in response to *Nmbr*-specific knockdown than *p65*-specific knockdown [19]. Therefore, we speculated that other pathways that might be involved in the *Nmbr*-mediated increase in [Ca^2+^]_i_ in addition to the p65/IL-6 pathway. Beyond NF-κB/p65, activator protein 1 (AP-1) is another key mediator associated with inflammation-induced PTB [14]. AP-1 is a transcription factor comprising members of the Fos and Jun families [20]. Moreover, c-Jun (Jun proto-oncogene, AP-1 transcription factor subunit), the most widely investigated protein of AP-1, is involved in the expression of various inflammatory genes by binding to their transcription factor binding sites [21]. c-Jun binds at the proximal *IL6* promoter to promote IL-6 generation in breast cancer cells [22]. The promoter of the human *PTGS2* (COX-2) gene contains several transcription factor binding sites, including AP-1 and NF-κB [23]. AP-1 can directly bind to *PTGS2* gene promoter to increase its expression in several cell types, including chondrosarcoma and tracheal smooth muscle cells [24,25], as well as human primary amnion cells [26]. Meanwhile, IL-6 and COX-2 expression was suppressed by AP-1 inactivation [27,28]. This evidence indicated that AP-1, in addition to p65, might be important to regulate the expression of IL-6 and COX-2 induced by NMB. Some studies have demonstrated a potential interaction between NF-κB and AP-1. The physical association of the leucine zipper domain of c-Jun and c-Fos with the Rel homology domain of the p65 subunit of NF-κB has been described, and this association enhances the transactivation of NF-κB-regulated genes [29]. In addition, Jun D co-operates with p65 to activate the proximal NF-κB site of the *CCND1* (cyclin D1) promoter [30]. However, a functional co-operation between NF-κB and AP-1 proteins in NMB-induced myometrial gene expression has never been investigated.

Taken together, these findings prompted us to investigate whether both c-Jun and p65 were involved in the regulation of COX-2 and IL-6 expression by NMB in human primary myometrial cells.

## Materials and methods

### Human subjects

Fifteen uterine smooth muscle specimens were collected from 15 pregnant women (singleton pregnancy, no complications, no premature rupture, or signs of infection) admitted to the Obstetrical Department of Xiangya Hospital of Central South University from August 2015 to May 2016. The average age of the pregnant women was 29.3 ± 3.6 years (25–34 years). The mean gestational time was 39^+6^ weeks (38^+4^ to 40^+3^ weeks). Planned cesarean delivery was carried out at terms in all 15 women because of pelvic stenosis, breech presentation, or certain other social-related factors.

### Sample collection

The experimental protocols were approved by the Ethics Review Committee of Central South University. Myometrial tissues (1 cm × 1 cm × 1 cm) were obtained from the upper edge of the uterine incision during lower segment cesarean section after fetus delivery and before oxytocin injection. Myometrial tissues were isolated and digested and dissociated and myometrial cells were collected and cultured according to our previous publication [19]. Myometrial cells could be amplified by passage culture. Cells at passages 3–6 were used for the experiments. All participating women provided written consent.

### Antibodies and reagents

Primary antibodies against total-p65, phospho-p65 (S536), total-c-Jun, phospho-c-Jun (Ser^63^/Ser^73^), and glyceraldehyde-3-phosphate dehydrogenase (GAPDH) were purchased from Cell Signaling Technologies (Danvers, MA, U.S.A.). Antibodies against NMBR weres purchased from Sigma–Aldrich (St. Louis, MO, U.S.A.). Antibodies against α-smooth muscle actin (α-SMA) were purchased from Abcam (Cambridge, U.K.). Horseradish peroxidase (HRP)–conjugated anti-rabbit or anti-mouse secondary antibodies were purchased from Cell Signaling Technologies (Danvers, MA, U.S.A.). An ECL-plus kit was purchased from Advansta (MenloPark, CA, U.S.A.). NMB was purchased from Bachem (Bubendorf, Switzerland). The human IL-6 enzyme-linked immunosorbent assay (ELISA) kit was purchased from Enzo Life Sciences (New York, NY, U.S.A.). Polybrene was purchased from Invitrogen (San Diego, CA, U.S.A.).

### Cell culture and treatment

Human primary myometrial cells were cultured in Dulbecco’s modified Eagle’s medium (DMEM), supplemented with 10% fetal bovine serum (FBS), penicillin (100 units/ml), and streptomycin (100 mg/ml) in 5% CO_2_ at 37°C in a humidified incubator. For cell treatment, 1 μM of NMB was added to the medium directly and incubated for various times (0.5, 1, 2, 4, or 6 h) before detection, while the same volume of medium was used as control.

### Measurement of IL-6 generation

Myometrial cells were treated with 1 μM of NMB or vehicle for the indicated times. The culture supernatant was collected to detect IL-6 using an ELISA kit according to the manufacturer’s instructions.

### qRT-PCR

Total RNA was isolated from untreated or NMB-treated cells using a Mini RNA Isolation II kit (Zymo Research, Irvine, CA, U.S.A.) according to the manufacturer’s instructions. CDNA was synthesized using SuperScript II reverse transcriptase (Invitrogen). Real-time polymerase chain reaction was performed using iTaq SYBR Green Supermix (Bio-Rad, Hercules, CA, U.S.A.) on an ABI ViiA™ real-time PCR detection system (Thermo Scientific, Waltham, MA, U.S.A.). The value of 2^–ΔΔ*C*^_t_ was used to determine the fold difference between samples. The following primers were used for *IL6*: Forward: 5′-TACATCCTCGACGGCATCTC-3′, Reverse: 5′-GCCATCTTTGGAAGGTTCAG-3′, *PTGS2*: Forward: 5′-GGTTGCTGGTGGTAGGAATG-3′, Reverse: 5′-TAAAGCGTTTGCGGTACTCA-3′, *ACTB* (encoding β-actin as a reference): Forward: 5′-CTCCATCCTGGCCTCGCTGT-3′, Reverse: 5′-GCTGTCACCTTCACCGTTCC-3′.

### Stable knockdown of p65 and c-Jun

Primary myometrial cells were infected with lentiviruses with shRNAs targeting p65 or c-Jun (Oligo-Engine, Seattle, WA, U.S.A.) and then selected with blasticidin (4 μg/ml; Invitrogen) for 2 weeks. The stable clones were pooled and used for further downstream experiments as indicated.

### Stable overexpression of p65 and c-Jun

Primary myometrial cells were infected with lentiviruses containing cDNA sequence of p65 or c-Jun (Oligo-Engine, Seattle, WA, U.S.A.) and then selected with puromycin (2 μg/ml; Invitrogen) for 3 weeks to generate stable clones, which were pooled and used for further downstream experiments as indicated.

### Western blotting

Protein samples were extracted from cells using radioimmunoprecipitation assay (RIPA) buffer [10 mM Tris/Cl (pH 8.0), 1 mM EDTA, 0.5 mM EGTA, 1% Triton X-100, 0.1% sodium deoxycholate, 0.1% SDS, and 140 mM NaCl]. Equivalent amounts of protein (30 μg of protein extract in each lane) were separated by 10% SDS/PAGE, transferred on to polyvinylidene fluoride (PVDF) membranes (Millipore Corporation, Bedford, MA, U.S.A.) and blocked with 5% non-fat milk/phosphate buffered saline-Tween 20 (PBST) solution for 1 h at room temperature. The membranes were then incubated with primary antibodies at 4°C overnight. The primary antibodies were detected using HRP–conjugated anti-rabbit or anti-mouse secondary antibodies and visualized using an ECL plus kit. GAPDH was used as the loading control. To normalize the Western blotting data, relative levels of phospho-p65, and phospho-c-Jun protein were normalized to the total p65 or c-Jun, respectively. Other non-phosphorylated proteins, like COX-2, were normalized to the internal control (GAPDH).

### Immunocytochemical staining

Isolated myometrial cells (2 × 10^6^ cells/dish) were cultured. Immunocytochemistry was used to examine the expression of NMBR in the surface and α-SMA in the cytoplasm. The cells were stained with anti-NMBR and anti-α-SMA polyclonal primary antibodies, followed by the appropriate HRP-linked secondary antibody. Cells were visualized under a microscope.

### Immunofluorescence staining

Cells fixed with 4% paraformaldehyde (PFA) solution, and permeabilized with Triton-X-100 were incubated with an anti-p65 (mouse) and anti-c-Jun (rabbit) antibodies overnight, after incubation with Alexa Fluor 488–conjugated anti-mouse secondary antibody (Abcam) and Alexa Fluor 647 anti-rabbit secondary antibody, 2-(4-amidinophenyl)-^1^H-indole-6-carboxamidine (DAPI) was used for nuclear staining. The final sections were examined under a confocal laser scanning microscope.

### Flow cytometry

Cells were suspended at 1 × 10^6^ cells/ml, and 5 μl of Annexin V and propidium iodide staining solution were added to 200 μl of the cell suspension. After the cells were incubated at room temperature for 15 min in the dark, stained cells were assayed and quantified using a FACSort Flow Cytometer (Beckman Coulter, Brea, CA, U.S.A.). Cell debris was excluded from the analysis using an appropriate forward light scatter threshold setting. Compensation was used whenever necessary.

### Statistical analysis

All quantitative data are expressed as mean ± standard deviation (SD) of at least three independent experiments. GraphPad Prism V software was used for statistical analyses. Differences between groups were compared using either Student’s *t* test or one-way analysis of variance (ANOVA) associated with Dunnett’s test. Correlation analysis between different indicators was carried out using Pearson correlation analysis. Statistical significance was defined as a *P*-value <0.05 (marked as *). Higher significance levels were set at *P*<0.01 (marked as **).

## Results

### Primary cell culture and identification

**Figure 1:**
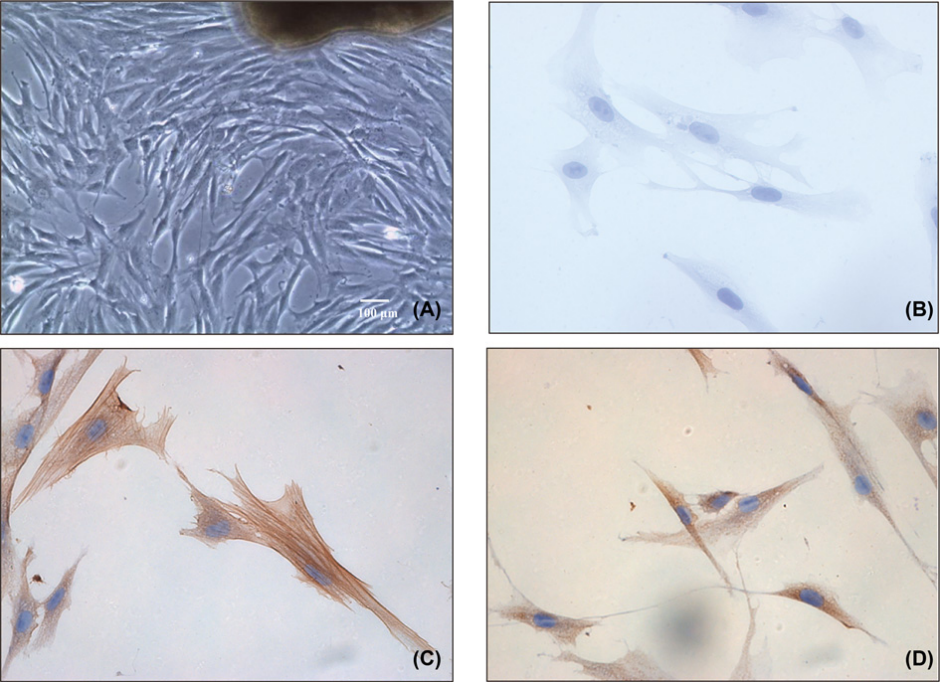
The cultured primary myometrial cells from term myometrium and their verification (**A**) Cultured primary myometrial cells were observed as long and fusiform in shape under inverted microscopy. At least five fields were counted. (**B**) The negative control for immunocytochemistry. At least five fields were counted. (**C**) The positive expression of α-SMA, as assessed by immunocytochemistry. At least five fields were counted. (**D**) The positive expression of NMBR, as assessed by immunocytochemistry. Original magnification ×40 (A) and ×400 (B–D). At least five fields were counted.

Cultured primary myometrial cells were long and fusiform in shape. Cell cloning was performed after 48 h of culture, the medium was first changed on day 5, and fusion was apparent in some clones 1 week later. The majority of cultured cells were observed to be long and fusiform or polygonal in shape under inverted microscopy (Figure 1A). Immunocytochemistry results showed α-SMA was expressed in abundance in the cytoplasm (Figure 1C) and NMBR was present in the membrane of the cultured myometrial cells (Figure 1D), compared with the negative control (Figure 1B). Taken together, these results showed that the cultured myometrial cells isolated from patients’ term myometrium demonstrated abundant levels of α-SMA and NMBR proteins.

### NMB up-regulates the expression of p-p65, p-c-Jun, IL-6, and COX-2 in primary myometrial cells

**Figure 2:**
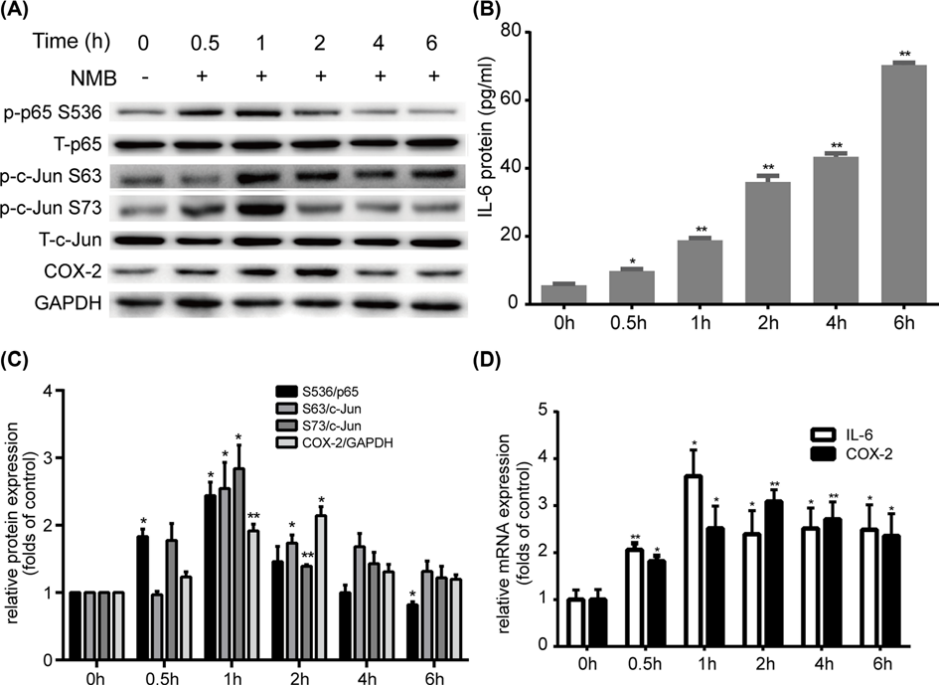
The effect of NMB on p65, c-Jun, COX-2 and IL-6 expression in cultured primary myometrial cells Cells were treated with 1 μM NMB for 0, 0.5, 1, 2, 4, and 6 h, and the protein levels were analyzed using Western blotting or ELISA, and the mRNA levels were analyzed by qRT-PCR. (**A**) Expression of total p65, p-p65 (Ser^536^), total c-Jun, p-c-Jun (Ser^63^, Ser^73^) and COX-2 was analyzed using Western blotting with GAPDH as a control standard. (**B**) IL-6 in the supernatants was quantified by ELISA. (**C**) The relative level of the COX-2 protein was normalized to that of the internal control (GAPDH). The relative levels of phospho-p65, and phospho-c-Jun protein were normalized to total p65 or c-Jun, respectively. (**D**) *IL6* and *PTGS2* mRNA levels were analyzed by qRT-PCR. Blots are representative. Data are expressed as mean ± SD of three independent experiments (*n*=3). *P<0.05; **P<0.01, against the control group.

Our previous study established the ideal dose of NMB in our system as 1 μM [19]. Therefore, we treated myometrial cells with 1 μM NMB for 0, 0.5, 1, 2, 4, or 6 h and then detected the expression of p65, c-Jun, IL-6, and COX-2 at the mRNA and protein levels. Our results showed that NMB treatment led to rapid phosphorylation of p65 and c-Jun without affecting their total levels (Figure 2A,C). Phosphorylation of p65 on Ser^536^, c-Jun on Ser^63^ and Ser^73^ were induced within 0.5 h of NMB treatment and reached a peak at 1 h (Figure 2A,C). *IL6* mRNA expression (Figure 2D) peaked at 1 h, and the IL-6 protein level (Figure 2B) increased dramatically following NMB treatment. COX-2 mRNA and protein levels (Figure 2A,C,D) peaked at 2 h. These findings demonstrated that NMB could significantly increase the levels of p-p65, p-c-Jun, IL-6, and COX-2 in primary myometrial cells.

### P65 and c-Jun play a vital role in NMB-induced COX-2 and IL-6 expression in primary myometrial cells

**Figure 3:**
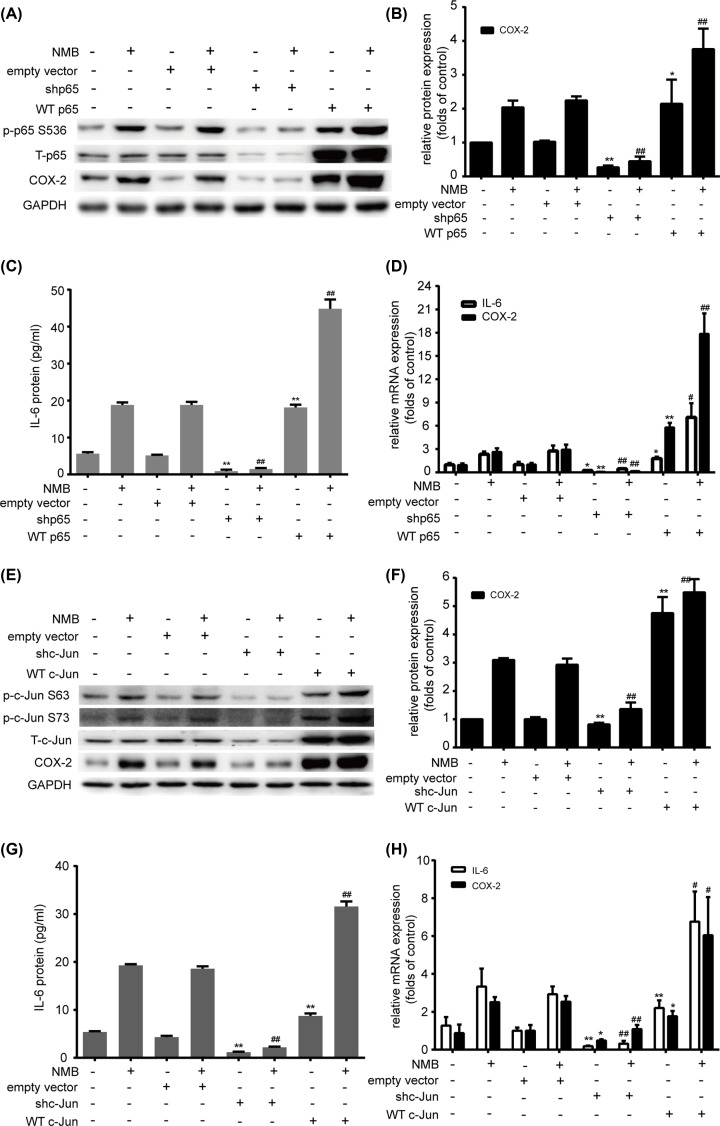
Induction of COX-2 and IL-6 by NMB is mediated via p65 and c-Jun (**A**–**D**) Primary myometrial cells were infected with lentiviruses with WTp65 overexpresssion or knockdown or the empty vector, and then treated with 1 μM NMB for 1 h. P-p65 (S536), p65, and COX-2 (A) levels were probed by Western blotting with GAPDH as an internal standard. The relative abundance of *PTGS2* was quantified (B). The release of IL-6 was measured by ELISA (C). *IL6* and *COX-2* mRNA induction was determined using qRT-PCR (D). (**E**–**H**) Primary myometrial cells were infected with lentiviruses with WT c-Jun overexpresssion or knockdown or the empty vector, and then treated with 1 μM NMB for 1 h. P-c-Jun (S63 and S73), c-Jun, and COX-2 (E) levels were analyzed using Western blotting with GAPDH as an internal standard. The relative abundance of COX-2 was quantified (F). The release of IL-6 was measured (G). *IL6* and *PTGS2* mRNA expression levels were determined using qRT-PCR (H). Blots are representative. Data are expressed as mean ± SD of three independent experiments (n=3). *P<0.05; **P<0.01, against the empty vector group; ^#^*P*<0.05; ^##^*P*<0.01, against the empty vector + NMB group.

To further determine whether p65 and c-Jun regulate COX-2 and IL-6 directly in myometrial cells, we depleted or overexpressed p65 or c-Jun. Efficacy of the knockdown or overexpression was verified by Western blotting analysis and qRT-PCR (Supplementary Figure S1). The results showed that p65 knockdown led to decreased COX-2 and IL-6 mRNA and protein levels with or without NMB treatment, compared with the empty vector group (Figure 3A–D). However, p65 overexpression resulted in increased COX-2 and IL-6 mRNA and protein levels, with or without NMB treatment, compared with the empty vector group (Figure 3A–D). Next, c-Jun was deleted or overexpressed to evaluate its function. Similarly, we found that c-Jun deletion evidently suppressed COX-2 and IL-6 mRNA and protein up-regulation with or without NMB treatment, compared with the empty vector group (Figure 3E–H), while c-Jun overexpression increased COX-2 and IL-6 mRNA and protein levels with or without NMB treatment, compared with the empty vector group (Figure 3E–H). Collectively, these results indicated the essential roles of p65 and c-Jun in NMB-induced expression of COX-2 and IL-6 in myometrial cells.

### Possible interaction between p65 and c-Jun in NMB-induced COX-2 and IL-6 expression in primary myometrial cells

**Figure 4:**
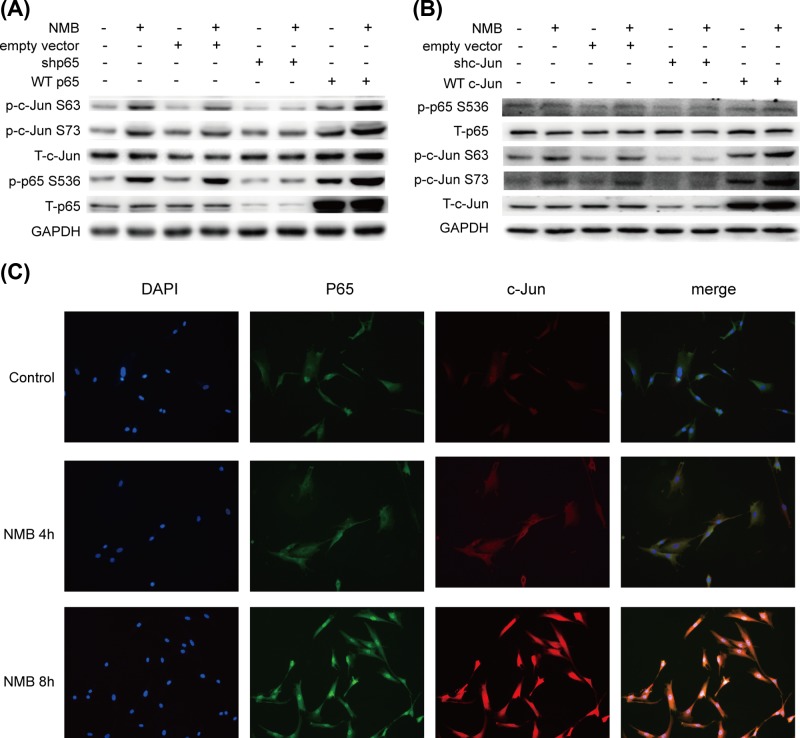
Potential interaction between NMB-induced expression of p65 and c-Jun (**A**) Primary myometrial cells were infected with lentiviruses with WTp65 overexpresssion or knockdown or the empty vector, and then treated with 1 μM NMB for 1 h. Levels of p65, c-Jun, p-p65 (S536), p-c-Jun (S63, S73) was analyzed by Western blotting with GAPDH as an internal standard. (**B**) Primary myometrial cells were infected with lentiviruses with WTc-Jun overexpresssion or knockdown or the empty vector, and then treated with 1 μM NMB for 1 h. Levels of p65, c-Jun, p-p65 (S536), p-c-Jun (S63, S73) were analyzed by Western blotting with GAPDH as an internal standard. Blots are representative of three independent experiments. (**C**) The cellular distribution of p65 (*green*, Alexa488) and c-Jun protein (*red*, Alexa647) at 0, 4, and 8 h after NMB treatment was visualized using confocal microscopy. Cell nuclei were stained with DAPI (*blue*). The images are representative of three independent experiments.

To our surprise, we found that level of phosphorylated c-Jun in the p65 knockdown group treated with or without NMB was significantly lower than that in the empty vector group (Figure 4A). While the level of phosphorylated c-Jun in the p65 overexpression group treated with or without NMB was significantly higher than that in the empty vector group (Figure 4A). Similarly, the level of phosphorylated p65 in the c-Jun knockdown group treated with or without NMB was significantly lower than that in the empty vector group (Figure 4B), and the level of phosphorylated p65 in the c-Jun overexpression group treated with or without NMB was significantly higher than that in the empty vector group (Figure 4B). Besides, we explored the cellular distribution of p65 and c-Jun using immunofluorescence. Both p65 and c-Jun translocated from the cytoplasm to the nucleus consecutively after NMB stimulation, and co-localization of p65 and c-Jun in the nucleus was observed following NMB treatment (Figure 4C), strongly suggesting an association between p65 and c-Jun.

### Correlations between NMB-induced phosphorylation of p65 or c-Jun and COX-2 or IL-6 protein levels in primary myometrial cells

**Figure 5:**
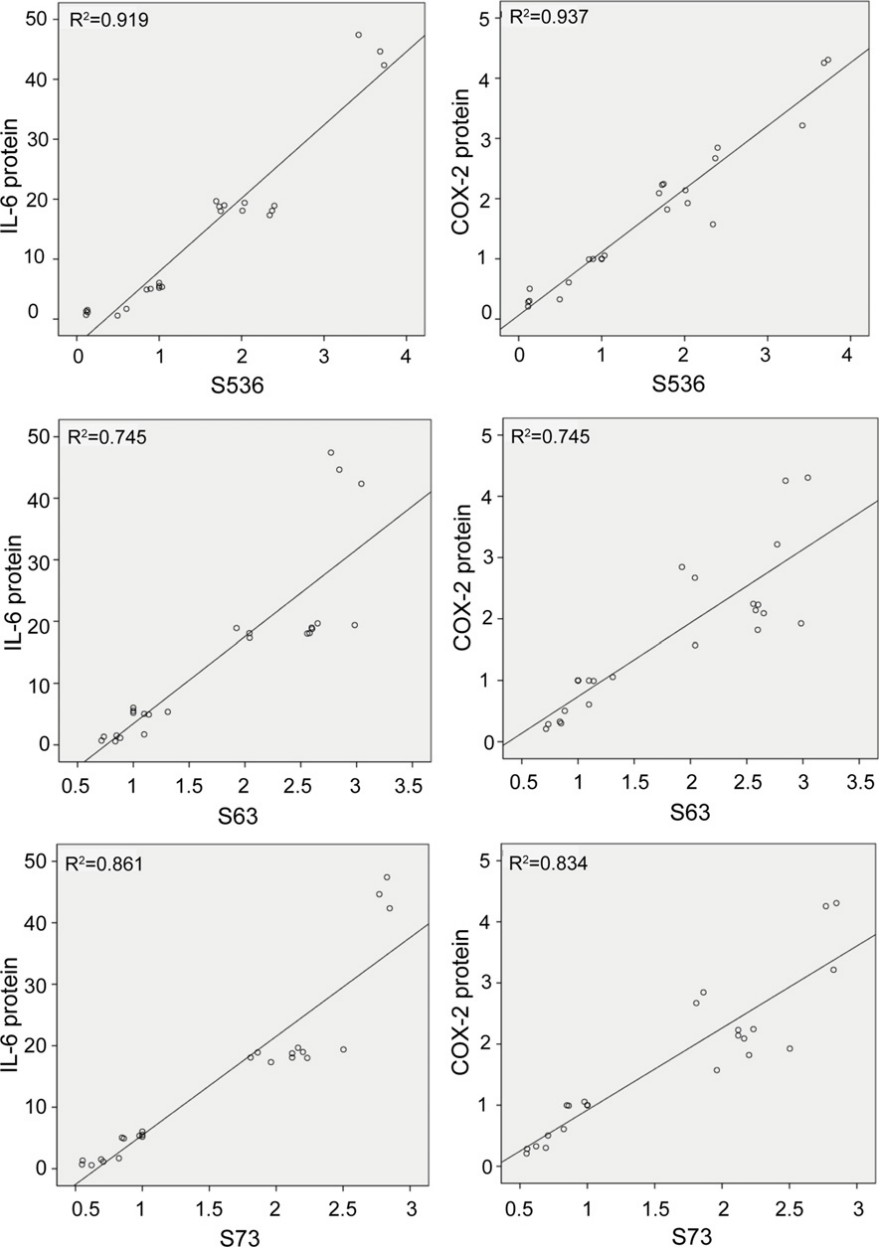
Correlations among NMB-induced expression of p-p65, p-c-Jun, COX-2, and IL-6 in primary myometrial cells Positive correlation was determined for p-p65 (S536) and IL-6 (*r* = 0.959, *P*<0.05), for p-p65 (S536) and COX-2 (*r* = 0.968, *P*<0.05), for p-c-Jun S63 and IL-6 (*r* = 0.863, *P*<0.05), for p-c-Jun S63 and COX-2 (*r* = 0.863, *P*<0.05), for p-c-Jun S73 and IL-6 (*r* = 0.928`, *P*<0.05), and for p-c-Jun S73 and COX-2 (*r* = 0.913, *P*<0.05).

As shown in Figure 5, positive correlations were detected between the level of phosphorylated p65 and the level of IL-6, and between the level of phosphorylated p65 and the level of COX-2 when induced by NMB (*r* = 0.959, *P*<0.05, and *r* = 0.968, *P*<0.05, respectively). Positive correlations were also observed between the level of phosphorylated c-Jun S63 and the level of IL-6 (*r* = 0.863, *P*<0.05), between the level of phosphorylated c-Jun S63 and the level of COX-2 (*r* = 0.863, *P*<0.05), between the level of phosphorylated c-Jun S73 and the level of IL-6 (*r* = 0.928, *P*<0.05), and between the level of phosphorylated c-Jun S73 and the level of COX-2 (*r* = 0.913, *P*<0.05) induced by NMB.

## Discussion

Many genes and associated pathways might contribute to preterm and term labor initiation [31,32]. In our initial cDNA microarray experiment, uterus fundus myometrial tissues were obtained from women undergoing cesarean section deliveries at term before the onset of labor and in labor. NMBR, but not the OTR or β_2_-adrenergic receptor, was differentially expressed [5]. The reason for this difference could be that the specimens were obtained from different parts of the uterus. OTR gene expression occurs in the cervix and lower uterine segment, with less pronounced changes in the uterine fundus [33]. The upper and lower regions of the human uterus function differently. The upper segment has a more contractile phenotype, contracting to push down and initiate delivery of the baby, while the lower segment maintains a more relaxed phenotype, promoting delivery of the baby through the lower segment of the uterus and cervix [34]. Therefore, we believe that NMBR will be a strong candidate target for tocolytics. However, the role of NMB/NMBR in regulating labor onset and the potential molecular mechanism are not fully defined.

Primary myometrial cell culture provides a suitable model to investigate the mechanism of NMB in labor initiation. The present study was conducted using cultures of primary myometrial cells isolated from women who chose planned cesarean section in our hospital. Immunochemical staining techniques demonstrated strong expression of α-SMA, thus confirming the myogenic origin of the cultured cells. The observed strong expression of NMBR in primary myometrial cells is consistent with our previous study [7].

Earlier studies reported increased concentrations of certain cytokines, most notably IL-6 in the serum and amniotic fluid of patients with preterm labor [35,36]. And the presence of IL-6 in the cervico-vaginal zone could predict preterm delivery [37]. Our previous study showed that NMB induced labor onset in mice associated with increased *IL6* mRNA expression [7]. In addition, IL-6 expression increased via the rela/p65 pathway in cultured mouse primary myometrial cells *in vitro* in response to NMB treatment [19]. This study confirmed that NMB could also significantly increase the expression of IL-6 at the mRNA and protein levels in human primary myometrial cells.

Prostaglandins (PGs) are believed to be involved in uterine contractions, cervical ripening and fetal membrane rupture during parturition [38]. COX-2 is the rate-limiting enzyme involved in PG synthesis. It increases in amnion cells and human myometrium in both term and preterm labor [11,18]. To the best of our knowledge, this is the first study to report that treatment of human primary myometrial cells with NMB could result in dramatic increase in COX-2 mRNA and protein levels. Therefore, COX-2, together with IL-6, is also likely to play a pivotal role in NMB-induced labor onset.

We next explored how NMB induced COX-2 and IL-6 expression in human primary myometrial cells. Through a combination of analyses, we demonstrated that activation of both p65 and c-Jun signaling are decisive factors in COX-2 and IL-6 up-regulation following NMB stimulation. In support of our observation, the *PTGS2* gene promoter has NF-κB and AP-1 binding sites, and activated p65 and c-Jun could induce *PTGS2* expression in several cell types, including human cervical cancer cells and human rheumatoid arthritis synovial fibroblasts [39,40]. In lipopolysaccharide (LPS)-induced inflammation, both NF-κB and AP-1 are activated and thus induce the expression of IL-6 and COX-2 [27,28]. Similarly, in our correlation analysis, we found that levels of phosphorylated p65 and c-Jun correlated positively with COX-2 and IL-6 expression. Therefore, the p65 and c-Jun pathways are hypothesized to be involved in NMB-induced up-regulation of COX-2 and IL-6. Besides, signal transducer and activator of transcription 3 (STAT3) were reported to bind to the *PTGS2* promoter region after cortisol stimulation in human amnion fibroblasts [41]. Whether other transcription factors like STAT3 mediate NMB-induced COX-2 induction in human myometrial cells require further investigation. Notably, as p65 or c-Jun knockdown led to cultured primary myometrial cells apoptosis (Supplementary Figure S2), we cannot rule out the possibility that this apoptosis could have a potential impact on the subsequent following experiments. NF-κB is a family of transcription factors that play critical roles in inflammation, immunity, cell proliferation, differentiation, and survival. Many miRNAs are transcribed by NF-κB and participate in the negative feedback loops to prevent sustained or excessive activation of NF-κB pathway [42]. Thus, as a key in physiological process, NF-κB not only activates inflammatory proteins like IL-6 but activates long non-coding RNA and other process in repair and cancer.

Evidence indicates that AP-1 can also exert a transactivating function independent of its binding to AP-1 sites via protein–protein interactions with other transcriptional regulators, including NF-κB and β-catenin [29,43]. A physical association of the basic leucine zipper regions of Jun/Fos with the Rel homology domain of the p65 NF-κB subunit resulting in enhanced transactivation of NF-κB-regulated genes has been previously demonstrated [29,30]. Interestingly, our current work predicted potential interaction between p65 and c-Jun in association with NMB action. This notion is supported by three lines of experimental evidence: (i) Either p65 or c-Jun knockdown could suppress downstream COX-2 and IL-6 expression in NMB treated cells. (ii) Knockdown of p65 could attenuate NMB-induced phosphorylation of c-Jun, and overexpression of p65 could increase the level of phosphorylated c-Jun induced by NMB. C-Jun knockdown or overexpression could affect NMB-induced phosphorylation of p65. (iii) High p65 co-occurrence with c-Jun was observed in the nuclei of NMB-treated myometrial cells. Thus, we believe that a similar mechanism contributes to *PTGS2* or *IL6* gene transactivation via the proximal NF-κB element of the promoter.

Our studies have important clinical implications. (1) In our pilot study, we established for the first time that NMBR is dramatically up-regulated during labor onset and sharply decreased after delivery, which indicates a close association with labor onset. (2) NMBR induced NF-κB and AP-1 activation. Thus, NMBR might up-regulate NF-κB, AP-1-responsive proinflammatory cytokines, chemokines, and MMPs in myometrial cells. NMBR may synergize with these mediators and induce labor onset. Therefore, targeting NMBR or developing NMBR antagonists might reduce the inflammation cascade and suppress uterine contraction, thus preventing preterm labor.

However, our study has some limitations. First, because of time and money limits, the sample size (n=3) for each dataset is relatively small, which is a weakness of our study. Second, although we demonstrated that NMBR activation could improve COX-2 and IL-6 expression by up-regulating p65 and c-Jun expression *in vitro*, we did not investigate whether such a phenomenon was replicated *in vivo*. Third, we established the ideal concentration of NMB as 1 μM in cell culture conditions based on our pilot study, which might not be consistent with physiological NMB concentrations during gestation and at parturition. Fourth, we reported that transcription factors p65 and c-Jun are involved in NMB-induced inflammatory cascade, but we cannot rule out the role of other transcription factors, such as STAT3 and Sp1, which are considered as typical factors involved in the inflammatory signaling pathway. Further studies are required to help fully understand the *in vivo* and *in vitro* involvement of the NMB/NMBR signaling pathway to provide robust evidence for NMBR as a target in anti-PTB therapy.

In summary, our study showed that in primary myometrial cells, NMB-induced COX-2 expression and IL-6 secretion were mediated through p65 and c-Jun activation. These results provide new insights into the mechanisms of NMB induced expression of COX-2 and generation of IL-6, with an aim of providing more evidence to explore NMBR as a novel therapeutic target.

## Supplementary Material

Supplementary Figures S1-S2

## Abbreviations

AP-1: activator protein 1
cDNA: complementary DNA
COX-2: cyclo-oxygenase 2
c-Jun: Jun proto-oncogene, AP-1 transcription factor subunit
EGTA: ethylene glycol-bis (beta-aminoethyl ether) - N, N, N’, N’-tetraacetic acid
ELISA: enzyme-linked immunosorbent assay
GAPDH: glyceraldehyde-3-phosphate dehydrogenase
HRP: horseradish peroxidase
IKK: IκB kinase
IL: interleukin
IκB: I kappa B
MMP: matrix metalloproteinase
NF-κB: nuclear factor κB
NMB: Neuromedin B
NMBR: NMB receptor
OTR: oxytocin receptor
PG: prostaglandin
PP2C: Protein Phosphatase 2C
PTB: preterm birth
qRT-PCR: Real-time Quantitative Reverse Transcription PCR
STAT3: signal transducer and activator of transcription 3
WIP1: The wild-type p53-induced Phosphatase 1
α-SMA: α-smooth muscle actin
