# Supplementary material for: Neuromedin B mediates IL-6 and COX-2 expression through NF-κB/P65 and AP-1/C-JUN activation in human primary myometrial cells
Source: Biosci Rep. 2019 Oct 11;39(10):BSR20192139. doi: 10.1042/BSR20192139 (PMC6822491; doi:10.1042/BSR20192139)
Supplement: Supplementary Figures S1-S2 [file BSR-2019-2139_supp.pdf]

SUPPLEMENTAL FIGURE LEGENDS

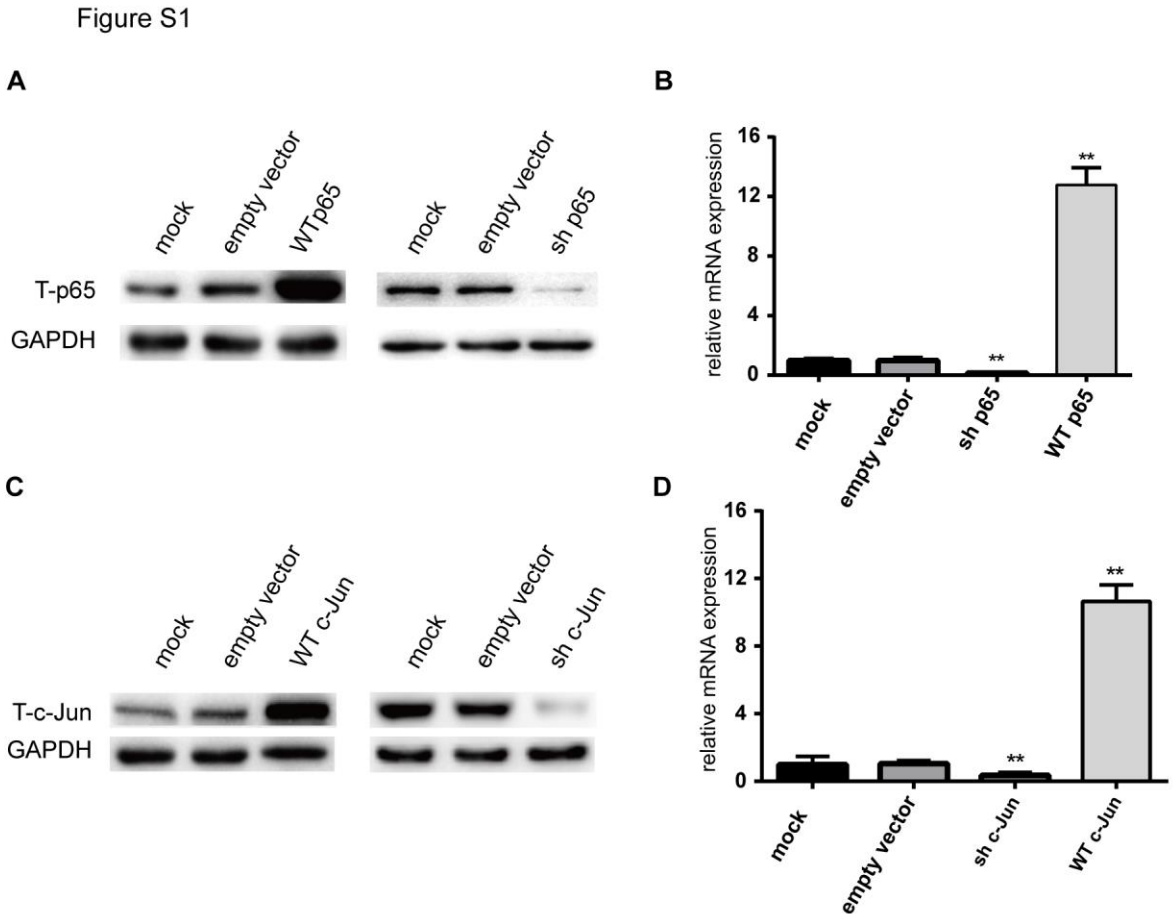

Figure S1. The efficiency of lentiviruses mediated p65 and c-Jun knockdown or overexpression in primary myometrial cells. A-B) The protein **(A)** and mRNA **(B)** levels of p65 in primary myometrialcells infected with lentiviruses with WTp65 overexpresssion or knockdown, respectively. **C-D)**The protein **(C)** and mRNA **(D)** levels of c-Jun in primary myometrial cells infected with lentiviruses with WTc-Jun overexpresssion or knockdown, respectively.Representative western blot results are shown. Data are expressed as mean ± SD of three independent experiments (n = 3). *P< 0.05; **P < 0.01, against the empty vector group.

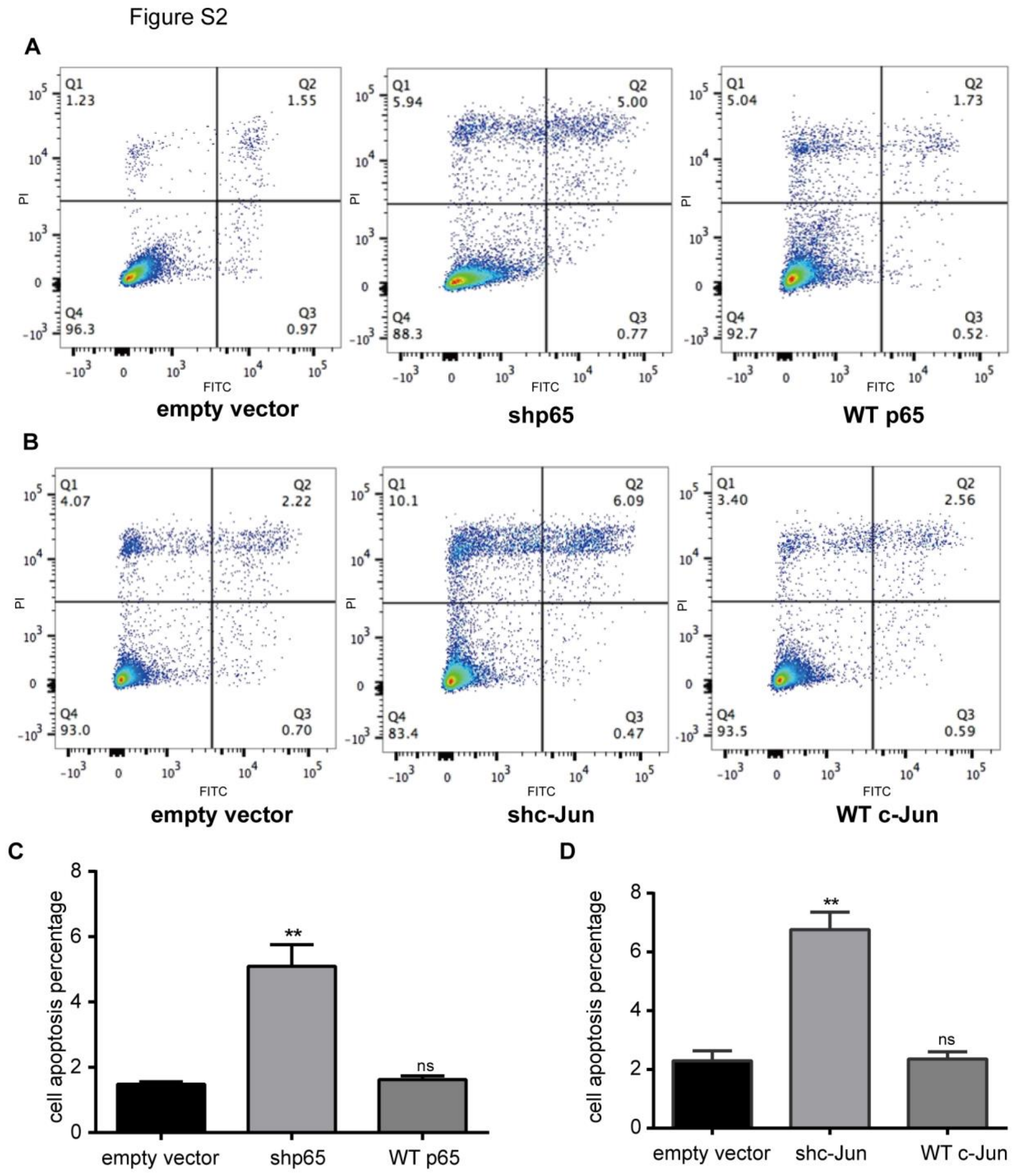

Figure S2. The Effect of p65 and c-Jun knockdown or overexpression on apoptosis of primary myometrial cells. A-B) Apoptosis of primary myometrial cells infected with lentiviruses with WTp65 (A) or WTc-Jun (B) overexpresssion or knockdown was determined by flow cytometry.Data are expressed as mean ± SD of three independent experiments (n = 3). **P*< 0.05; ***P*< 0.01, against the empty vector group.
